# Cardiovascular safety of transcutaneous spinal cord stimulation in cervical spinal cord injury

**DOI:** 10.1016/j.neurot.2025.e00528

**Published:** 2025-01-31

**Authors:** Soshi Samejima, Raza N. Malik, Jennifer Ge, Lucas Rempel, Kawami Cao, Sameer Desai, Claire Shackleton, Anahita Kyani, Parisa Sarikhani, Jessica M. D'Amico, Andrei V. Krassioukov

**Affiliations:** a International Collaboration on Repair Discoveries, Faculty of Medicine, University of British Columbia, Vancouver, BC, Canada; b Division of Physical Medicine and Rehabilitation, Department of Medicine, University of British Columbia, Vancouver, BC, Canada; c Department of Rehabilitation Medicine, University of Washington, Seattle, WA, USA; d School of Population and Public Health, University of British Columbia, Vancouver, BC, Canada; e GF Strong Rehabilitation Centre, Vancouver Coastal Health, Vancouver, BC, Canada; f ONWARD Medical, Lausanne, Switzerland; g Glenrose Rehabilitation Hospital, Alberta Health Services, Edmonton, Canada; h Department of Medicine, University of Alberta, Edmonton, Canada

**Keywords:** Spinal cord injuries, Transcutaneous spinal cord stimulation, Cardiovascular function, Rehabilitation, Safety assessment

## Abstract

This study evaluated whether cervical transcutaneous spinal cord stimulation (tSCS) in conjunction with rehabilitation on upper extremity function alters blood pressure regulation in individuals with cervical spinal cord injury. This study is a secondary analysis of the Up-LIFT trial, a prospective single-arm multicenter trial designed to evaluate the safety and efficacy of tSCS in conjunction with rehabilitation (tSCS ​+ ​rehab) on upper extremity function in individuals with chronic cervical spinal cord injury. Utilizing this large data set obtained from 60 individuals across 14 international sites, we compared blood pressure and heart rate measurements obtained before, during and after each training session during both the wash-in Rehab alone period and the tSCS ​+ ​rehab period of the trial. Blood pressure and heart rate were recorded during each session throughout the protocol in all participants. Sessions of tSCS ​+ ​rehab did not cause significant changes in blood pressure or heart rate compared to Rehab alone (p ​> ​0.05). Further, blood pressure medications did not have an effect on these cardiovascular responses to tSCS (p ​> ​0.05). This study supports the safety profile of cervical tSCS paired with rehabilitation in individuals with cervical spinal cord injury. The lack of adverse effects on blood pressure and heart rate during the intervention, together with the previously reported clinically meaningful improvements in upper extremity strength and function strongly supports the utility of tSCS in this patient population. Further work is required to elucidate potential long-term effects of targeted tSCS on cardiovascular function in people with spinal cord injury.

## Introduction

Cervical spinal cord injury (SCI) disrupts crucial communication between the spinal cord circuits and the supraspinal control centers, resulting in a pernicious cascade of severe impairments in motor function, including upper extremity function [1,2]. Furthermore, disruption of supraspinal control to the autonomic spinal cord circuits often causes cardiovascular dysregulation including low resting blood pressure (BP), sudden drops in BP known as orthostatic hypotension (OH), and uncontrolled rises in BP known as autonomic dysreflexia (AD) [3,4]. OH and AD, clinically defined as greater than 20 ​ ​mmHg drops and rises in SBP, are essentially the result of either insufficient or excessive vasoconstriction, respectively [5]. Recurrent BP fluctuations also place individuals with SCI at increased risk for further cardio- and cerebro-vascular accidents [6]. Cardiovascular dysfunction negatively impacts morbidity and mortality after cervical SCI [7,8]. Despite the numerous pharmacological agents available to manage abnormal cardiovascular control in individuals with SCI, they have significant limitations including delayed onset of effect and various side effects [4].

Epidural spinal cord stimulation (eSCS) has demonstrated promising results in promoting recovery of motor and autonomic function in individuals with SCI [[9], [10], [11], [12], [13], [14], [15], [16], [17], [18]]. However, eSCS is an invasive and costly procedure involving surgical implantation of electrode arrays [19]. Transcutaneous spinal cord stimulation (tSCS) is a novel and non-invasive approach that allows electrical stimulation to be delivered to targeted spinal cord regions with minimal pain or discomfort [20]. The evidence of tSCS for the recovery of upper extremity function is emerging and was previously limited to pilot studies with small sample sizes [[21], [22], [23], [24]]. More recently, however, the pivotal Up-LIFT trial (NCT04697472) assessed the safety and effectiveness of tSCS at the cervical spinal cord in 60 individuals with chronic, cervical SCI. Using an earlier generation of the ARC^EX^ system, cervical tSCS delivered in conjunction with rehabilitation safely improved arm and hand function in individuals with chronic, cervical SCI [25].

Despite the clinically meaningful improvements reported across multiple studies, recent publications have raised the safety concern that spinal cord stimulation might lower the threshold for AD and induce AD after SCI [26,27], especially in individuals with cervical SCI such as those enrolled in the Up-LIFT Trial. This report explores the impact of cervical tSCS on cardiovascular parameters before, during and after each training session during each phase of the trial (rehab only vs tSCS ​+ ​rehab) to better understand the safety profile of cervical tSCS on BP regulation in individuals with chronic, cervical SCI.

## Methods

### Study design

The Up-LIFT trial evaluated the safety and effectiveness of programmed non-invasive spinal cord stimulation (ARC^EX^ Therapy, ONWARD Medical, Inc., Boston, MA, NCT04697472) in conjunction with rehabilitation, using an earlier generation of the ARC^EX^ System. The trial enrolled 65 participants with chronic, cervical incomplete SCI (American Spinal Injury Association Impairment Scale [AIS] B, C, and D) across 14 centers. A full participant inclusion/exclusion criteria list can be found in Moritz et al. 2024. All participants provided written informed consent. A two-month “run-in phase” of upper extremity rehabilitation was followed by a two-month “treatment phase”, combining tSCS and rehabilitation (tSCS ​+ ​rehab). The rehabilitation program was defined to ensure homogeneity across sites. This program involved approximately 1 ​h of training, 3–4 times per week, with functional task categories that included repetitive activities of gross upper extremity movement, isolated finger movements, simple and complex pinch, and grasping activities. Each participant performed 1–2 exercises within each category during each treatment session [25]. The stimulation was delivered through a pair of round surface electrodes placed between the spinous processes at one segment above and one below the injury site. Two larger reference rectangular electrodes were placed bilaterally over the iliac crests (89 ​% of sessions) or the clavicles to disperse the electrical current over a larger surface area for tissue safety and to minimize discomfort. The device administered biphasic or monophasic waveforms at 30 ​Hz burst frequency, pulse width of 1 ​ms, and a 10 ​kHz carrier frequency. The stimulation waveforms were set based on which configuration produced the most pronounced arm and hand movements. The stimulation amplitude was below the motor threshold and did not induce movements or discomfort. During the tSCS ​+ ​rehab phase, stimulation was delivered continuously during the 60-min rehabilitation session. Full details of stimulation parameters can be found in the recent report [28] and are reported according to the spinal cord stimulation minimum reporting standards [29]. Briefly, biphasic stimulation was utilized most frequently in 83 ​% of all sessions, with average current amplitudes 39.3 ​± ​25.5 ​mA and 52.4 ​± ​31.0 ​mA for mono- and bi-phasic stimulation waveforms, respectively. Furthermore, the average current densities of each active electrode on the neck, used across all participants, were 2.44 ​± ​1.59 ​μC/cm^2^ for the monophasic 1 ​ms pulse and 3.26 ​± ​1.93 ​μC/cm^2^ for the biphasic 1 ​ms pulse. Sixty subjects completed ≥24 sessions (12 sessions/month) during each phase of the trial.

In addition to the prespecified primary and secondary safety and effectiveness outcomes [25], blood pressure (BP) and heart rate (HR) were collected pre, mid and post each training session during each phase of the trial. Each study site used their available traditional oscillometric BP and HR monitoring devices, such as the Carescape Dinamap V100 (GE Healthcare, USA) and Welch Allyn VSM 300 Patient Monitor (Welch Allyn, USA). Of special note, the mid-training BP/HR measurements during the tSCS ​+ ​rehab phase of the trial were performed during active stimulation. The findings presented in this manuscript result from a secondary analysis of this data set. In this secondary analysis, the primary outcome was systolic blood pressure (SBP), and secondary outcomes were diastolic BP (DBP) and HR. The incidence rate of autonomic dysreflexia (AD) per treatment phase was also calculated by assessing changes in SBP. The incidence rate of high SBP change per phase was calculated by using the frequency of SBP increase during the intervention divided by all sessions in all participants. The severity of SBP change was classified into four categories: ≥20 ​mmHg to <30 ​mmHg, ≥30 ​mmHg to <40 ​mmHg, ≥40 ​mmHg to <50 ​mmHg, and ≥50 ​mmHg.

### Statistical analysis

All statistical analyses were conducted using the R 4.3.2 statistical programming software [30]. Descriptive statistics, including mean, median, confidence intervals and standard deviation, frequency, and percentages, as appropriate, were used to summarize participant characteristics, systolic blood pressure (SBP), diastolic blood pressure (DBP), and heart rate (HR).

To estimate differences in cardiovascular control between the treatment phases, generated Linear Mixed Effect (LME) models were used for each outcome variable that included the fixed effects of Treatment Phase (rehab only vs tSCS ​+ ​rehab) and timepoint (Pre, Mid, Post training session), the random intercept of participant, and random slopes for Treatment Phase following an unstructured correlation pattern using the lme4 R package [31]. The referents for Treatment Phase, and Timepoint were the Rehab only condition, and pre-session recordings. The model controlled for Age, Sex, and AIS by including these continuous and categorical variables. The referent for AIS was the AIS B subgroup.

Tables of estimates, confidence intervals, p values, and random effects were created and visualized using the sjPlot package. The model fit was assessed using information criteria [Akaike Information Criteria (AIC)], where smaller values represent a better fitting model. All models were estimated using Maximum Likelihood. Our final model was selected by first including the interaction terms assessing the two-way interaction between treatment phase and timepoint. If no two-way interactions were observed (p ​> ​0.05), the predictors were entered into the model without interacting with other predictors. The Wald Test and AIC were used to identify the best-fitting model.

Model diagnostics was performed for all LMEs using the DHARMa [32] and performance package [33] on each outcome's final model. The QQ (quantile-quantile) plot of residuals and a plot of residuals against predicted values were also evaluated to test the assumptions of normality and homogeneity of variance.

Lastly, the incidence rate of autonomic dysreflexia during each treatment phase of the trial was not normally distributed (Shapiro-Wilk test p ​< ​0.05), thus non-parametric statistics were used to evaluate this outcome measure. Specifically, the Wilcoxon signed rank test was used to evaluate the differences in the incidence rate of AD during each phase of the study.

## Results

**Fig. 1:**
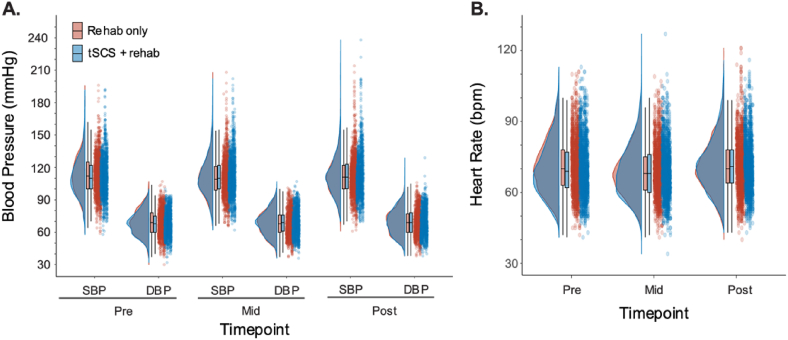
Blood pressure and heart rate for each treatment phase and timepoint during treatment sessions. Raincloud plots are shown to highlight the probability density, box plots and raw data for pre, mid, and post session systolic blood pressure (**A**, SBP), diastolic blood pressure (**A**, DBP), and heart rate (**B**, HR) for Phase 1 (Rehab alone, Red) and Phase 2 (tSCS ​+ ​rehab, Blue).

Baseline demographics of the patient population are presented in Table 1. Pre, Mid and Post SBP, DBP, and HR are reported in Fig. 1 and Table 2.

**Table 1:** Participant demographics and injury characteristics.

Variable	Population completed the trial (n ​= ​60)
Age, Years, mean (SD)	42.7 (15.5)
Sex, no. (%)	Female: 10 (16.7), Male: 50 (83.3)
Neurological level of injury, no. (%)	C2: 9 (15.0), C3: 3 (5.0), C4: 16 (26.7), C5: 13 (21.7), C6: 13 (21.7), C7: 6 (10.0), C8: 0 (0)
American spinal injury association impairment scale (AIS), no. (%)	AIS B: 9 (15.0), AIS C: 28 (46.7), AIS D: 13 (38.3)
Time since injury, Years, mean (SD)	6.1 (7.5)

**Table 2:** Cardiovascular characteristics in all participants (N ​= ​60).

Treatment Phase	Phase 1: Rehab alone			Phase 2: tSCS ​+ ​rehab		
Timepoint	Pre	Mid	Post	Pre	Mid	Post
SBP, mmHg	112.7 ​± ​17.8	110.7 ​± ​18.0	112.2 ​± ​19.0	111.5 ​± ​17.7	112.1 ​± ​18.6	113.2 ​± ​19.8
DBP, mmHg	68.9 ​± ​11.6	68.3 ​± ​11.3	69.3 ​± ​11.6	68.2 ​± ​10.7	69.0 ​± ​11.1	69.5 ​± ​11.8
HR, bpm	71.2 ​± ​11.2	71.2 ​± ​11.3	69.0 ​± ​10.6	71.6 ​± ​11.3	70.1 ​± ​11.7	68.8 ​± ​11.1

### Effects of tSCS on blood pressure and heart rate

To specifically assess the acute effects of multiple sessions of tSCS ​+ ​rehab, we examined SBP, DBP and HR recorded at three timepoints; at baseline before applying the intervention (Pre), middle of the intervention (Mid) and right after the intervention (Post) for each training session (Table 2, Fig. S2). We compared the change in measurements during the tSCS ​+ ​rehab treatment sessions (mid-pre and post-pre, Fig. S3) to those obtained during the rehab-only phase. There were no interaction effects between treatment phase and timepoint. BP and HR were stable throughout each therapy session and were not adversely affected by tSCS (Table 3, all p ​> ​0.45). Although our statistical models indicate a significant main effect of timepoint on SBP, DBP, and HR (Table 3, p-values <0.05), this was not attributed to the addition of tSCS, but rather likely an exercise-mediated effect with the minimal estimated differences indicating no clinically meaningful changes in BP or HR [34].

**Table 3:** Linear mixed effects models (LMEs) fitted to estimate the relationship between treatment phase and timepoint on systolic BP (SBP), diastolic BP (DBP), heart rate (RATE) while including the covariates of age, injury type, and sex.

*Predictors*	SBP			DBP			HR		
	*Estimates*	*95 ​% CI*	*p*	*Estimates*	*95 ​% CI*	*p*	*Estimates*	*95 ​% CI*	*p*
(Intercept)	88.53	72.57–104.5	**<0.001**	50.21	41.32–59.10	**<0.001**	70.98	61.83–80.13	**<0.001**
Phase [tSCS ​+ ​rehab]	0.33	−0.90–1.56	0.598	0.03	−0.77–0.82	0.946	−0.28	−1.01–0.45	0.456
Timepoint [mid]	−0.73	−1.29–−0.17	**0.011**	0.14	−0.22–0.49	0.453	−0.8	−1.16–−0.43	**<0.001**
Timepoint [post]	0.53	−0.03–1.09	0.063	0.83	0.47–1.18	**<0.001**	−2.54	−2.90–−2.17	**<0.001**
Age	0.4	0.18–0.62	**<0.001**	0.28	0.15–0.40	**<0.001**	−0.12	−0.25–0.01	0.065
AIS [C]	0.48	−9.52–10.48	0.926	−0.2	−5.76–5.36	0.945	−0.82	−6.55–4.92	0.78
AIS [D]	2.67	−7.66–13.00	0.612	1.79	−3.95–7.53	0.541	0.83	−5.09–6.75	0.784
Sex [Male]	4.14	−4.96–13.24	0.373	5.57	0.51–10.63	**0.031**	7.45	2.23–12.66	**0.005**
**Random effects**									
Residual variance	122.43			49.69			52.01		
Random intercept variance	178.3			58.9			57.69		
Random slope variance	20.19			8.55			6.89		
Random effects covariance	−0.13			−0.29			−0.06		
ICC	0.6			0.53			0.54		
Observations	8986			8986			8988		
Marginal R^2^/Conditional R^2^	0.121/0.645			0.175/0.615			0.108/0.586		

Referent: Rehab only, Pre, AIS B, Female; ICC, Intraclass Correlation Coefficient. Significant *p* values (i.e., <0.05) are bolded.

**Fig. 2:**
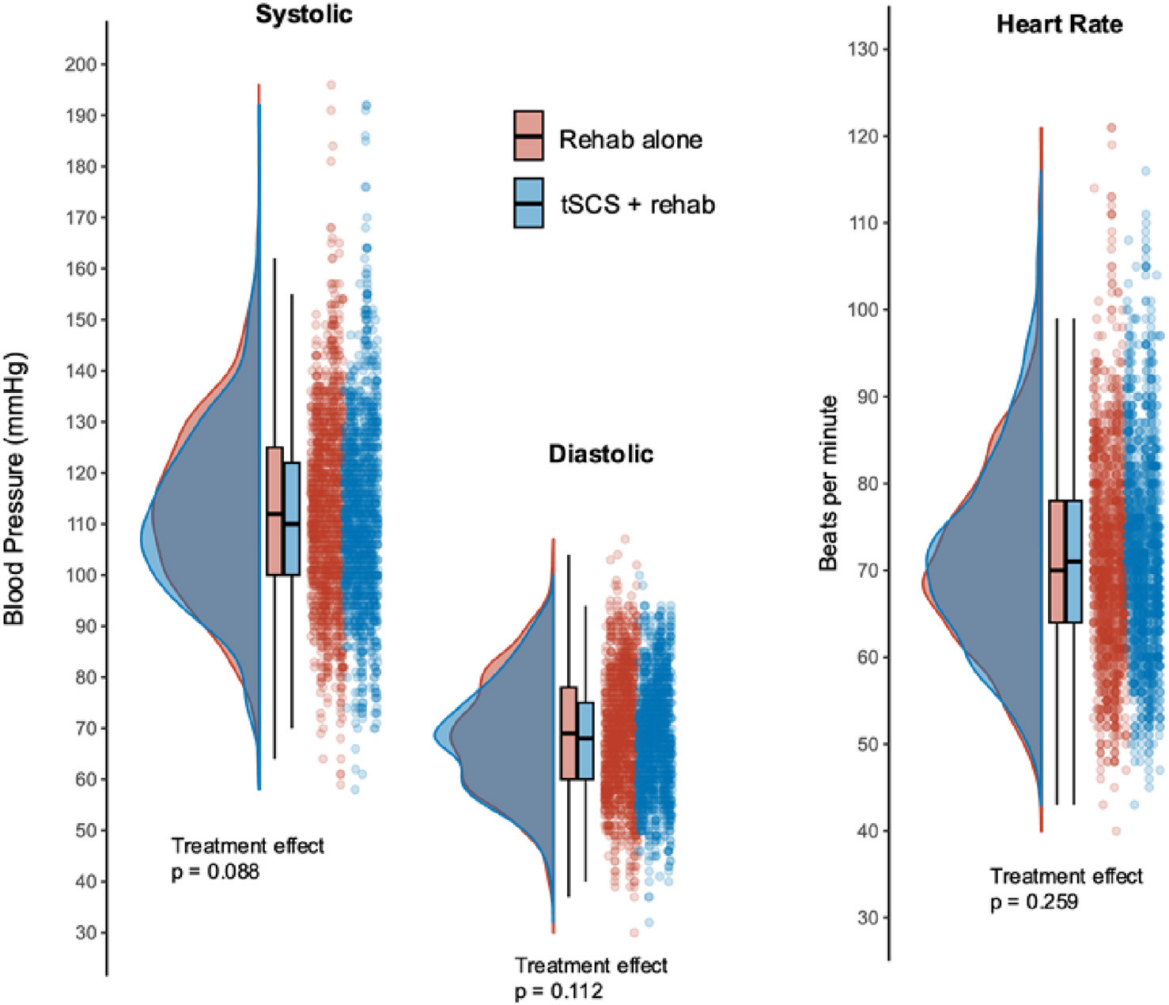
Pre-session blood pressure and heart rate for each phase of therapy. Raincloud plots are shown to highlight the probability density, box plots and raw data for pre-session systolic blood pressure (SBP), diastolic blood pressure (DBP), and heart rate (HR) during Rehab alone, (Red) and during tSCS ​+ ​rehab, (Blue).

To further assess whether continued tSCS ​+ ​rehab facilitated long-term changes in baseline BP and HR, pre-session SBP, DBP, and HR during the Rehab alone and the tSCS ​+ ​rehab phases were compared (Fig. 2). Our statistical model confirmed that the therapy phase had no effect on pre-session BP and HR (Table 4, all p ​> ​0.08), indicating that tSCS similarly did not induce significant long-term changes over time in BP and HR (Fig. S2).

**Table 4:** Linear mixed effects models (LMEs) fitted to estimate the relationship between treatment phase and systolic BP (SBP), diastolic BP (DBP), heart rate (RATE) while including the covariates of age, injury type, and sex.

*Predictors*	SBP			DBP			HR		
	*Estimates*	*95 % CI*	*p*	*Estimates*	*95 ​% CI*	*p*	*Estimates*	*95 ​% CI*	*p*
(Intercept)	88.95	73.97–103.92	**<0.001**	51.03	42.51–59.56	**<0.001**	72.89	63.72–82.07	**<0.001**
Phase [tSCS ​+ ​rehab]	−1.29	−2.77–0.19	0.088	−0.8	−1.78–0.19	0.112	0.41	−0.30–1.13	0.259
Age	0.35	0.14–0.55	**0.001**	0.24	0.13–0.36	**<0.001**	−0.15	−0.28–−0.02	**0.02**
AIS [C]	0.34	−9.03–9.70	0.944	−0.3	−5.62–5.01	0.91	−1.53	−7.29–4.22	0.601
AIS [D]	3.26	−6.41–12.93	0.508	2.01	−3.48–7.50	0.473	1.15	−4.79–7.09	0.704
Sex [Male]	7.5	−1.02–16.02	0.085	6.93	2.09–11.76	**0.005**	6.74	1.50–11.97	**0.012**
**Random effects**									
Residual variance	119.67			49.72			52.75		
Random intercept variance	168.17			60.51			56.60		
Random slope variance	24.50			11.05			3.82		
Random effects covariance	−0.32			−0.46			0.02		
ICC	0.57			0.52			0.53		
Observations	2997			2997			2998		
Marginal R^2^/Conditional R^2^	0.118/0.623			0.168/0.602			0.112/0.580		

Referent: Exercise only, AIS B, Female; ICC, Intraclass Correlation Coefficient. Significant *p* values (i.e., <0.05) are bolded.

### Effects of tSCS on incidence of autonomic dysreflexia

**Fig. 3:**
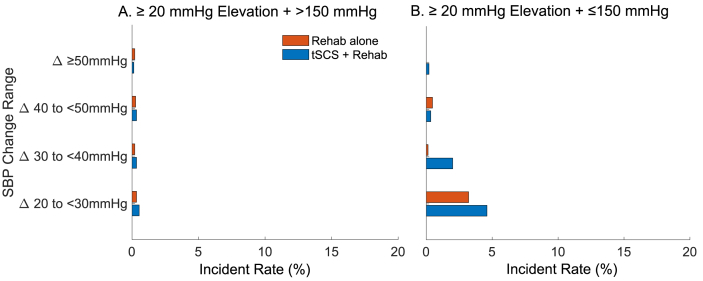
The incidence rate of systolic blood pressure (SBP) change ≥ +20 ​mmHg. The change in mid-session SBP relative to pre-session SBP across all sessions is depicted. The incidence rate per treatment phase (Rehab alone (red) vs tSCS ​+ ​rehab (blue)) was calculated from all treatment sessions during each phase in all participants. (A) The incidence rates of increases in SBP resulting in AD requiring monitoring were similar between the two treatment phases. (B) The incidence rates of ≥ +40 ​mmHg SBP change inducing ​≤ ​SBP 150 ​mmHg were similar between the two treatment phases. The incidence rate of < +40 ​mmHg SBP change increased during tSCS ​+ ​rehab.

Utilizing this data set, we explored further the incidence rate of episodes of AD during the two treatment phases. Adverse event reporting during the Up-LIFT trial previously reported 4 incidents of AD across 2 participants during tSCS ​+ ​rehab, with all cases mild, and only 2 incidents probably related to stimulation [25]. However, it is possible that individuals with SCI experience ‘silent’ AD [35], and therefore these occurrences may not have been captured during adverse event reporting during the trial as it appears without recognized symptoms. Utilizing BP measurements obtained during each training session, we assessed the change in SBP mid-session relative to the pre-session SBP and defined AD as a change greater than 20 ​ ​mmHg in SBP. We further characterized fluctuations in SBP into severe (requiring management, Fig. 3A) or non-severe AD (Fig. 3B) using a cutoff of 150 ​ ​mmHg in SBP. There was no difference in the incidence rate of severe AD between the Rehab alone and tSCS ​+ ​rehab phases of the trial (3.4 ​± ​7.3 ​% and 4.0 ​± ​6.0 ​%, p-value ​= ​0.07). On the other hand, the incidence rate of non-severe AD significantly increased during tSCS ​+ ​rehab when compared to Rehab alone (4.4 ​± ​7.7 ​% and 6.9 ​± ​8.1 ​%, p-value <0.001, Wilcoxon signed rank test). Further analysis of this finding revealed that in these instances (Δ20-40 ​ ​mmHg and overall SBP <150 ​ ​mmHg) the mean pre-session SBP was 99 ​± ​12 ​mmHg, with 78 ​% of instances occurring in hypotensive individuals (<110 ​ ​mmHg) indicating that at least a significant proportion of increased SBP incidents, while meeting the clinical definition of AD, normalized SBP in these patients.

### Effects of antihypertensive and antihypotensive medications

Twenty-four of 60 participants (40 ​%) reported taking one or more BP medications during the trial. For analysis purposes, BP medications were defined as medications prescribed with the intention to increase or decrease BP, excluding medications that may incidentally influence BP as an unintended effect. From the 24 participants on BP medications, 15/24 (62.5 ​%) were prescribed antihypotensive medications, 7/24 (29.2 ​%) were prescribed antihypertensive medications, and 2/24 (8.3 ​%) were prescribed both types of medication (Fig. S1). The usage of antihypertensive or antihypotensive medications did not alter changes in SBP during Rehab alone (without medications: 110.9 ​± ​19.1 ​ ​mmHg, with medications: 113.3 ​± ​17.0 ​ ​mmHg) or tSCS ​+ ​rehab (without medications: 111.0 ​± ​19.6 ​ ​mmHg, with medications: 114.2 ​± ​17.2 ​ ​mmHg, Table 5, Table 6).

**Table 5:** The impact of blood pressure medications on cardiovascular responses.

Study phase	Phase 1: Rehab alone			Phase 2: tSCS ​+ ​rehab		
Timing	Pre	Mid	Post	Pre	Mid	Post
Participants without blood pressure medications (N ​= ​36)						
SBP, mmHg	111.7 ​± ​18.1	109.8 ​± ​18.6	111.1 ​± ​20.3	109.9 ​± ​17.7	111.0 ​± ​19.4	112.0 ​± ​21.5
DBP, mmHg	68.9 ​± ​11.8	68.0 ​± ​11.5	69.1 ​± ​12.0	67.7 ​± ​10.7	68.7 ​± ​11.6	69.2 ​± ​12.3
HR, bpm	72.4 ​± ​11.3	73.3 ​± ​11.4	71.1 ​± ​10.9	73.0 ​± ​11.1	72.1 ​± ​12.4	70.7 ​± ​11.8
Participants on blood pressure medications (N ​= ​24)						
SBP, mmHg	114.2 ​± ​17.4	112.0 ​± ​16.9	113.7 ​± ​16.8	113.9 ​± ​17.4	113.8 ​± ​17.3	114.9 ​± ​16.8
DBP, mmHg	68.9 ​± ​11.3	68.8 ​± ​10.9	69.4 ​± ​10.9	68.8 ​± ​10.7	69.4 ​± ​10.5	69.9 ​± ​10.9
HR, bpm	69.5 ​± ​10.9	68.0 ​± ​10.5	65.9 ​± ​9.3	69.6 ​± ​11.3	67.3 ​± ​10.0	66.1 ​± ​9.3

**Table 6:** Linear mixed effects models (LMEs) fitted to estimate the relationship between medications and systolic BP (SBP) and heart rate (RATE) while including the covariates of, age, injury type, and sex.

*Predictors*	SBP			DBP			HR		
	*Estimates*	*CI*	*p*	*Estimates*	*CI*	*p*	*Estimates*	*CI*	*p*
(Intercept)	87.54	71.61–103.46	**<0.001**	49.93	41.01–58.84	**<0.001**	72.24	63.41–81.08	**<0.001**
Meds [on Meds]	3.68	−3.59–10.95	0.321	1.25	−2.93–5.44	0.557	−4.78	−8.78–−0.77	**0.019**
Phase [tSCS ​+ ​rehab]	−0.13	−1.70–1.44	0.87	−0.25	−1.27–0.77	0.631	−0.38	−1.32–0.56	0.425
Timepoint [mid]	−0.73	−1.29–−0.17	**0.011**	0.14	−0.22–0.49	0.453	−0.8	−1.16–−0.43	**<0.001**
Timepoint [post]	0.53	−0.03–1.09	0.063	0.83	0.47–1.18	**<0.001**	−2.54	−2.90–−2.17	**<0.001**
Age	0.41	0.19–0.63	**<0.001**	0.28	0.16–0.40	**<0.001**	−0.13	−0.25–−0.00	**0.045**
AIS [C]	−0.99	−11.21–9.23	0.85	−0.82	−6.52–4.89	0.779	0.84	−4.83–6.51	0.771
AIS [D]	2.4	−7.82–12.63	0.645	1.69	−4.02–7.40	0.562	1.04	−4.64–6.71	0.72
Sex [Male]	4.11	−4.89–13.12	0.371	5.57	0.54–10.60	**0.03**	7.5	2.51–12.50	**0.003**
**Random effects**									
Residual variance	122.43			49.69			52.01		
Random intercept variance	175.20			58.46			52.84		
Random slope variance	19.87			8.44			6.87		
Random effects covariance	−0.14			−0.29			−0.05		
ICC	0.59			0.53			0.52		
Observations	8986			8986			8988		
Marginal R^2^/Conditional R^2^	0.134/0.646			0.181/0.615			0.145/0.58		

Referent: Not on Meds, Exercise only, Pre, AIS B, Female; ICC, Intraclass Correlation Coefficient. Significant p values (i.e., <0.05) are bolded.

## Discussion

This secondary analysis aimed to assess the safety of tSCS in conjunction with rehabilitation on cardiovascular function in a cohort of 60 participants with chronic cervical SCI. Our analysis indicates that cervical tSCS combined with rehabilitation did not alter BP or HR when compared to rehabilitation alone.

A recent study has highlighted growing safety concerns with electrical stimulation techniques, such as tSCS, citing an increased incidence rate of AD [26,36]. In contrast, there is evidence showing cervical spinal cord stimulation can lead to positive changes in cardiovascular control in individuals with chronic pain [[37], [38], [39]], heart conditions [40,41], and SCI [22]. Our investigation found that cervical tSCS did not alter BP or HR during treatment sessions with tSCS when compared to rehabilitation alone, nor did it induce lasting changes in resting BP over time. The lack of significant effect of cervical tSCS on cardiovascular function during the Up-LIFT Trial is not surprising as the area of localization of the majority of sympathetic preganglionic neurons controlling blood vessels lies within the T6-T12 vertebral levels [42]. A recent work in significantly fewer participants with SCI elucidated that there could be potential safety concerns with cervical tSCS related to autonomic dysfunction [43].

In addition, the analysis of AD incidence rate revealed that real-time cervical tSCS could elevate SBP but in a controlled and safe manner, keeping SBP below 150 ​mmHg. When SBP exceeded 150 ​mmHg, a threshold deemed clinically significant and warranting pharmacological management and close monitoring [44], the incidence rate of AD during active stimulation did not significantly differ from that observed with rehabilitation alone. This finding is similar to previous studies demonstrating real-time mid-thoracic tSCS elevated SBP during orthostatic challenge [45,46]. These findings underscore the safety of cervical tSCS as a neuromodulation strategy when combined with rehabilitation for individuals with cervical SCI. Nonetheless, it is important to note that stimulation of thoracic and lumbosacral regions of the spinal cord could lead to more robust cardiovascular effects compared to cervical tSCS in people with SCI, which may result in increased susceptibility to AD risks [47]. However, we would also like to acknowledge that thoracic and lumbosacral tSCS could control elevations and drops in SBP by modulating stimulation intensity [46,48].

As indicated above, the current AD definition, an increase in SBP >20 ​mmHg, alone may not reflect all clinical scenarios. We showed that tSCS could increase SBP by more than >20 ​mmHg during exercise to the normotensive range in individuals with hypotension, which can facilitate training [49]. Our findings suggest that assessing the safety of interventions aimed at mitigating vascular-related risks requires consideration of additional factors beyond the current AD definition and guidelines, such as underlying conditions (e.g., hypotension) and concurrent activities (e.g., exercise) [44,50,51].

There are some limitations in this secondary analysis. Primarily, BP and HR were monitored intermittently using oscillometric BP devices at only three time points per training session. The limited BP monitoring potentially overlooked transient BP fluctuations during therapy. Additionally, the absence of electrocardiogram (ECG) monitoring prevented the evaluation of tSCS effects on heart rhythm, including potential arrhythmias and changes in QRS complex duration throughout the protocol. Secondly, the participant cohort included only nine individuals with AIS B injury severity (15 ​%), who are more likely to exhibit severe cardiovascular dysfunctions compared to those with more preserved descending pathways [52]. Despite no observed correlation between AIS classification and BP in the trial cohort, a more balanced distribution of injury severity could reveal further insights. Lastly, the current data set could not provide insights into the direct effects of systematic manipulation of stimulation parameters on cardiovascular control given the nature of the trial design and its focus on recovery of upper extremity function. However, prior analysis on the incidence of “possibly-related” adverse device events which could include incidents of AD or significant changes in BP or HR demonstrated a distribution of mono- or bi-phasic waveforms that mirrored the overall distribution during the trial [25]. Additionally, the stimulation amplitudes utilized during these occurrences were lower than the average amplitudes reported for each waveform during the trial and significantly below the maximum amplitudes implemented during the Up-LIFT Trial [28]. Nonetheless, further systematic investigation is needed to assess the effect of stimulation configurations including electrode location, stimulation intensity, and waveform on cardiovascular outcomes.

In summary, our data indicates that cervical tSCS is safe, as evidenced by the safety endpoints established in both the primary analysis and this additional secondary analysis of cardiovascular function. The ARC^EX^ Therapy could be safely performed without inducing severe AD in people with cervical SCI. This safety assessment supports wider clinical use of cervical tSCS paired with rehabilitation for improving strength, function and quality of life following cervical SCI, which guides clinicians and researchers. Further research is necessary to thoroughly investigate the effectiveness and underlying mechanisms of paired tSCS and rehabilitation for cardiovascular regulation in this patient population.

## Data sharing statement

All data generated and analyzed during this study are included in this published article and in the online-only materials, further inquiries can be directed to the corresponding author.

## Ethic statement

The trial and recruitment materials were approved by institutional review boards or ethics committees at each clinical trial site as well as central approval from the Advarra institutional review board.

## Author contributions

The study was conceived and designed by S.S. and A.V.K. Data was collected by S.S and A.V.K and each site PIs. Data was analyzed by S.S., R.N.M, A.K., P.S., and J.M.D. S.S. wrote the manuscript with support from R.N.M, A.K., and J.M.D. All authors provided critical revision of the manuscript and final approval of the version to be published.

## Funding/Support

This study was supported by ONWARD Medical. A.V.K. holds Endowed Chair in rehabilitation medicine, 10.13039/501100005247University of British Columbia, and his lab is supported by funds from the Canadian Institutes for 10.13039/100005622Health Research, Canada Foundation for Innovation and BC Knowledge Development Fund, 10.13039/501100000334International Spinal Research Trust, 10.13039/100012436Rick Hansen Foundation, PRAXIS Spinal Cord Institute, Craig H. 10.13039/100005191Neilsen Foundation, 10.13039/100008191Wings for Life Research Foundation, and US 10.13039/100000005Department of Defense. S.S. is supported by 10.13039/100007080Paralyzed Veterans of America Fellowship, 10.13039/100008191Wings for Life Spinal Cord Research Foundation, 10.13039/100009713Foundation for Physical Therapy Research, Craig H. 10.13039/100005191Neilsen Foundation, Mission Yogurt Fund, and Morton Cure Paralysis Fund. RNM is supported by fellowships from the 10.13039/100007080Paralyzed Veterans of America, 10.13039/501100000245Michael Smith Health Research BC, and the Canadian Training Platform for Trials Leveraging Existing Networks. C.S is supported by the 10.13039/100007080Paralyzed Veterans of America Fellowship and Canadian Institute for 10.13039/100005622Health Research Fellowship. 10.13039/501100009051SS, RNM, and CS are also supported by the 10.13039/100012436Rick Hansen Foundation.

## Declaration of competing interest

A.K. and P.S. are employees of ONWARD Medical. J.M.D. is a paid consultant of ONWARD Medical. The remaining authors reported no conflict of interest.
