# Supplementary material for: Cardiovascular safety of transcutaneous spinal cord stimulation in cervical spinal cord injury
Source: Neurotherapeutics. 2025 Jan 31;22(2):e00528. doi: 10.1016/j.neurot.2025.e00528 (PMC12014404; doi:10.1016/j.neurot.2025.e00528)
Supplement: Multimedia component 1 [file mmc1.docx]

**Supplementary information**


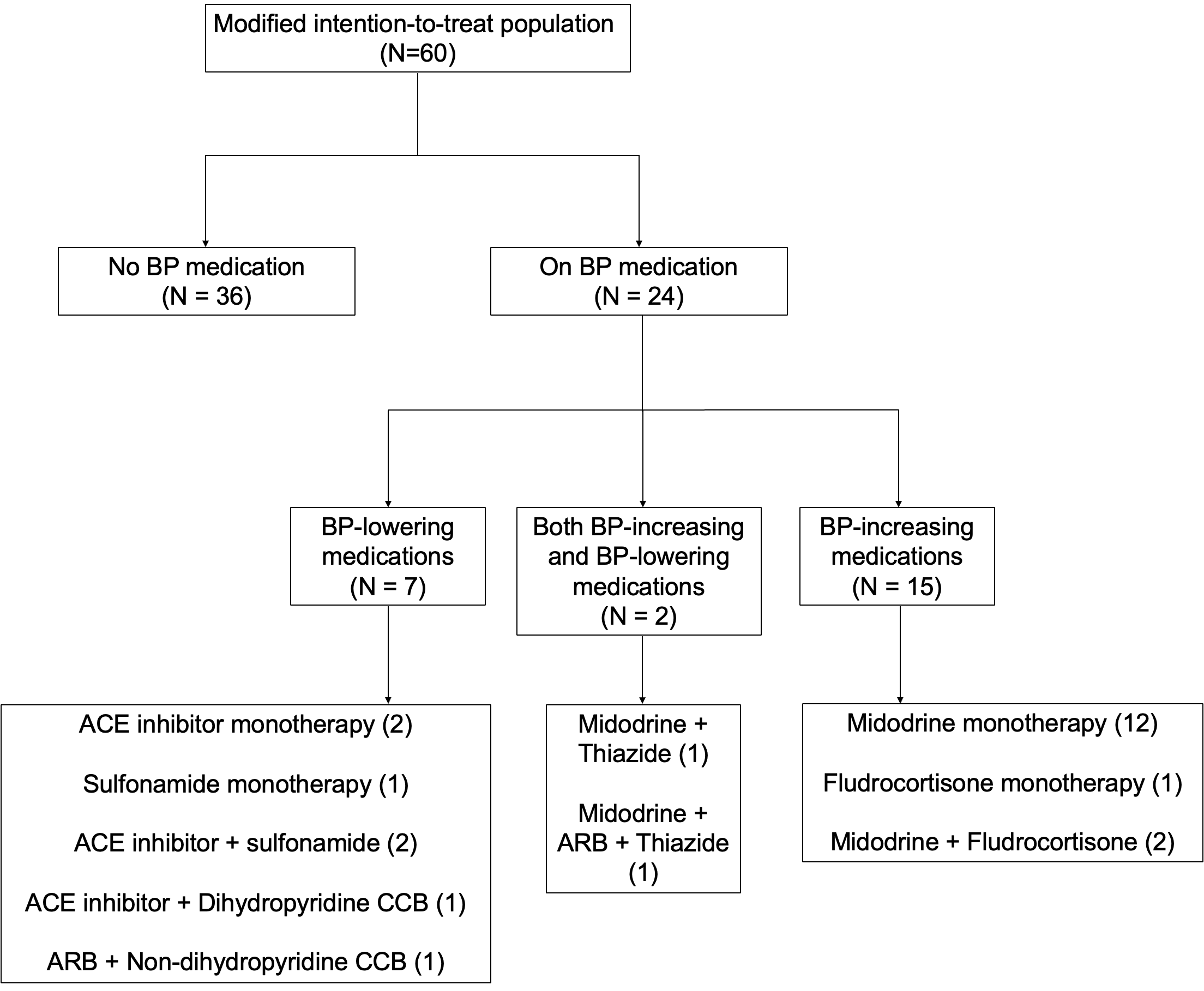


**Figure S1.** Medication Category with The Number of Participants (No.). Abbreviation: ACE: Angiotensin Converting Enzyme, ARB: Angiotensin Receptor Blockers, BP: Blood Pressure, CCB: Calcium Channel Blocker.


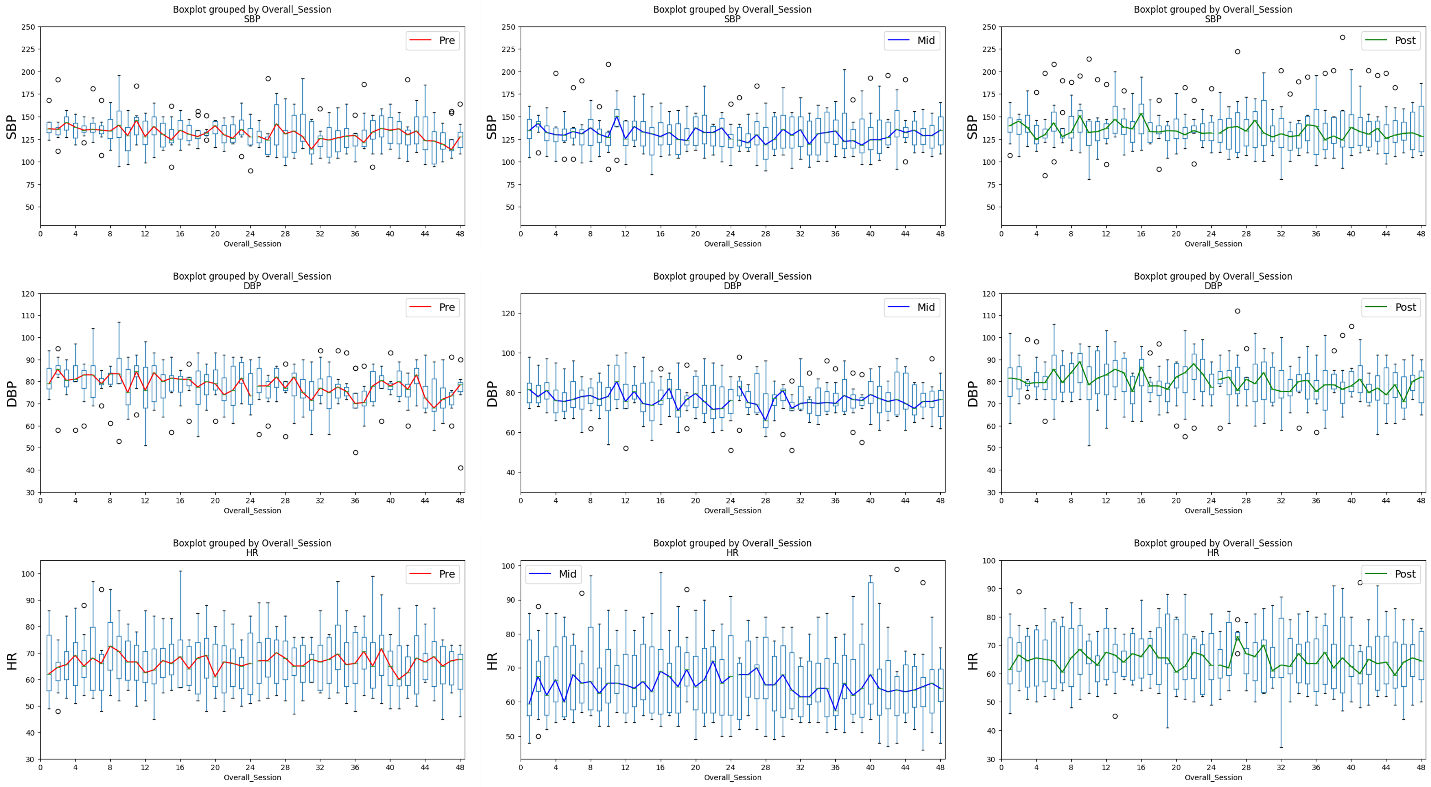


**Figure S2.** Raw SBP, DBP and Heart Rate recorded across all training sessions and at each timepoint (pre-, mid- and post-session). Of note, mid-session recording during the second phase of the trial (sessions 25 onwards) were obtained during active tSCS.


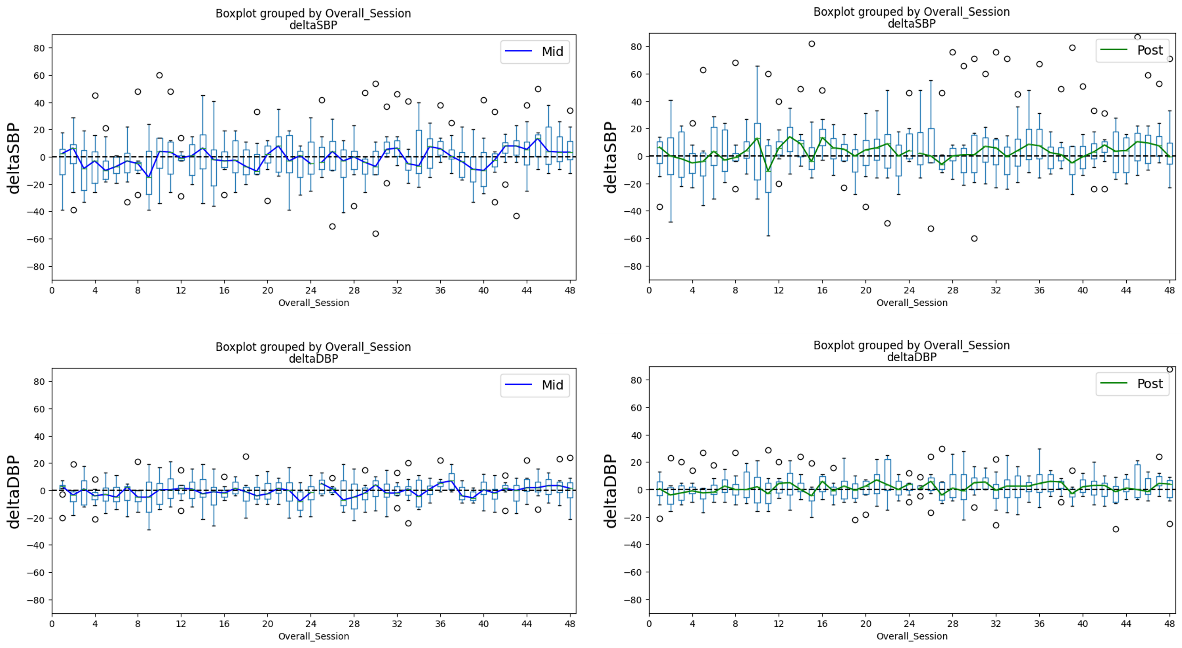


**Figure S3.** Change in SBP, DBP and Heart Rate across all training sessions and at each timepoint (mid-pre and post-pre). Of note, mid-session recording during the second phase of the trial (sessions 25 onwards) were obtained during active tSCS.
